# Psychiatric burden in hereditary cancer syndromes: Implications for genetic counseling and mental health Care

**DOI:** 10.1192/j.eurpsy.2026.11100

**Published:** 2026-07-31

**Authors:** N. Bouayed Abdelmoula, B. Abdelmoula, R. Rhaiem, S. Sellami, S. Aloulou, W. Smaoui, K. Trigui

**Affiliations:** LR23ES07 Genomics of Signalopathies aat the service of Precision Medicine, Medical University of Sfax, Sfax, Tunisia

## Abstract

**Introduction:**

Hereditary cancer syndromes exert significant psychological stress on mutation carriers and their families, manifested by distress, anxiety and depression.

**Objectives:**

Understanding this psychiatric burden is essential for comprehensive genetic counseling and effective psychosocial interventions.

**Methods:**

We carried out a review of the narrative literature to synthesize recent evidence on psychological concerns in people affected by hereditary cancer syndromes.

**Results:**

Clinically relevant distress affects about two-thirds of carriers, with almost half suffering from anxiety and more than a third reporting depression. Women, especially those with a personal history of cancer, have higher rates of depressive symptoms. The main contributing factors include uncertainty about the prognosis of the disease, prophylactic treatment decisions, family communication problems and care-related stress. These psychiatric symptoms can compromise the quality of life and compliance with monitoring protocols. Family members also suffer psychological impacts due to genetic risk and the burden of care.

**Conclusions:**

The psychiatric burden of hereditary cancer syndromes is multifaceted and influenced by biological, psychological and social factors. The integration of mental health support into genetic counseling frameworks is essential to cope with emotional distress, improve adaptation and improve medical adherence. Early identification of psychological distress can mitigate the risk of demoralization and suicidal ideation in vulnerable people. Holistic care in hereditary cancer syndromes requires coordinated genetic and psychiatric services to manage the complex emotional challenges faced by patients and families, ultimately improving psychosocial and clinical outcomes.

**Disclosure of Interest:**

None Declared

